# Validation of the Fatigue Impact Scale in Multiple Sclerosis Patients in Serbia

**DOI:** 10.3390/brainsci14080825

**Published:** 2024-08-17

**Authors:** Olivera Tamaš, Marija Kovačević, Aleksandra Dobrodolac, Nada Rašuo Bosnić, Žužana Tot Šari, Livija Despenić, László Vécsei, Krisztina Bencsik, Tatjana Pekmezović, Jelena Drulović

**Affiliations:** 1 Neurology Clinic, University Clinical Center of Serbia, 11000 Beograd, Serbia; adobrodolac@gmail.com (A.D.); drulovicjelena@gmail.com (J.D.); 2 Medical Faculty, University of Belgrade, 11000 Belgrade, Serbia; marijakov8@gmail.com (M.K.); pekmezovic@orion.rs (T.P.); 3 General Hospital Subotica, 24000 Subotica, Serbia; nada.rasuo.bosnic@gmail.com (N.R.B.); zuzana.totsari@gmail.com (Ž.T.Š.); suneuro@gmail.com (L.D.); 4 Department of Neurology, Albert Szent-Györgyi Faculty of Medicine and Clinical Centre, University of Szeged, H-6725 Szeged, Hungary; vecsei.laszlo@med.u-szeged.hu (L.V.); krisztina.bencsik@gmail.com (K.B.); 5 Institute of Epidemiology, Faculty of Medicine, University of Belgrade, 11000 Belgrade, Serbia

**Keywords:** fatigue, questionnaire, validation, multiple sclerosis

## Abstract

Fatigue is one of the most frequent complaints of patients with multiple sclerosis (MS). The Fatigue Impact Scale (FIS), one of the 30 available fatigue questionnaires, is commonly applied because it evaluates multidimensional aspects of fatigue. The chief objectives of this study were to validate FIS and evaluate the psychometric properties of MS patients in Serbia. One hundred and twenty-one (121) MS patients and one hundred and twenty-two (122) age-, gender- and education-matched healthy control (HC) subjects completed the FIS and the Beck Depression Inventory. Internal consistency of the FIS subscales was determined using Cronbach’s Alpha Coefficient. Test/retest reliability with an intra-class correlation coefficient (ICC) for each FIS subscale was performed. The total FIS score and subscale scores showed statistically significant differences between the MS patients and the HC subjects in both FIS sessions. Cronbach’s Alpha was 0.966. All ICCs were statistically significant (*p* < 0.05). The Serbian version of this instrument may be useful as a clinical measure for fatigue and functionality in patients with MS.

## 1. Introduction

Fatigue is one of the most common symptoms associated with multiple sclerosis (MS) and was described decades ago [1,2]. Mills and Young previously defined fatigue as “reversible, motor and cognitive impairment with reduced motivation and desire to rest, either appearing spontaneously or brought on by mental or physical activity, humidity, acute infection and food ingestion. It can occur at any time but is usually worse in the afternoon. In MS, fatigue can be daily, has usually been present for years and has greater severity than any premorbid fatigue” [3]. Although there is no unique definition of fatigue, it is usually defined as an overwhelming feeling of tiredness or exhaustion due to the lack of both mental and physical energy that interferes with one’s normal daily activities [4,5]. Fatigue may be acute, lasting up to six weeks, or chronic fatigue that is present for at least half the time continuously for a period of over six weeks. In addition, fatigue may be classified into primary fatigue that has no apparent cause and is most likely specific to MS. Secondary fatigue is due to some other underlying mental disorder, systemic condition, hormone or electrolyte disturbances or drug use [6,7]. Additionally, in patients with MS, fatigue has to occur independently from muscle weakness and depression [8]. A substantial number of studies aim to investigate the underlying mechanisms and the pathophysiology of fatigue in patients with MS. It is believed that there is no single pathway implicated. Rather, a number of different central and peripheral mechanisms have been elucidated that can contribute to fatigue in MS. Based on this, central fatigue is characterized by reduced performance on cognitive tasks, disturbances in motivation and other central nervous system (CNS) effects, while peripheral fatigue refers to muscular fatigability [9]. Overall, it has been described that fatigue in MS is a multidimensional symptom that spans various elements from different areas [10]. 

One can distinguish two qualities of fatigue. Firstly, the fatigue trait refers to the general predisposition to experience fatigue. This is quantified by fatigability, which may, in turn, either be perceived or objective and measured. Then, there is fatigue state, which indicates the current condition, which is dynamic and can be measured at different points in time [6,11].

Fatigue affects up to 80% of MS patients, and a more recent study found an annual incidence of fatigue in MS of up to nearly 30% [6]. MS fatigue has been described to be severe in 65% to 70% of patients [1,12,13], with over half of patients grading fatigue as the worst or one of their worst symptoms [14]. In persons with MS, fatigue may be present from the early stages of the disease and it may persist continuously throughout the disease course [15]. Fatigue reduces the quality of life and significantly impacts an individual’s mental and physical wellbeing, as well as daily activities including their ability to work and their social functioning [6,16]. Quality of life in MS is fundamentally influenced by different symptoms, predominantly by fatigue, presumably through indirect pathways and physical disability as well as by different mental factors, such as self-efficacy [17].

Therefore, it is important to clinically detect, assess and manage fatigue in patients with MS. Assessment of fatigue in MS heavily relies on subjective reports and patients’ introspective abilities [6]. A variety of different clinical tools may aid in uniformly assessing different aspects of fatigue in each case. A number of different questionnaires and tests are available. Amongst the first ones, the fatigue severity scale was validated in the late 1980s by Krupp [18]. The Fatigue Impact Scale (FIS) [19] is one of the 30 available fatigue questionnaires. Both the FIS and the shorter version, the modified FIS, are widely used. While the modified FIS is short and does not require much time for completion, it has been found that total scores are not sufficiently valid [20]. More detailed questionnaires, particularly the FIS, inquire about different items from various domains, providing a more integrative approach. It is commonly applied because it evaluates multidimensional aspects of fatigue. Ultimately, it assesses the impact of fatigue and offers a detailed description of the patient’s functional status. As of now, there is no standardized and validated scale in Serbian language inquiring about fatigue in persons with MS.

The objectives of this study were to validate the FIS and evaluate the psychometric properties after translation and cultural adaption of this scale. 

## 2. Materials and Methods

The FIS was chosen out of a variety of questionnaires because the 40 questions evaluate fatigue as a complex symptom: 10 items pertain to cognitive, 10 to physical and 20 to social domains [19] (Copyright 1991, J. D. Fisk, P.G. Ritvo and C. J. Archibald). Each item is scored between 0 (no problem) and 4 (severe problem). The total score is expressed as a continuous scale ranging between 0 and 160 points. The original FIS was developed in English. The translation and adaptation into the Serbian language were performed through an initial independent translation of the complete questionnaire into Serbian, followed by an independent translation of the translated document back to English and the revision of the items that did not match the original English document. The final phase of the translation was a consensus meeting, which included translators, patients and health care professionals. They gave suggestions and helped rephrasing certain items in the questionnaire in a way that made them more understandable to persons of different educational backgrounds.

In the Serbian version, several items had to be reviewed, most notably the word “fatigue”. In Serbian, there are two similar words, one of which describes tiredness, while the other is more associated with the meaning of fatigue. The latter was accepted for use in all items. In item 4, the direct translation of “moody” was changed to a more descriptive construction similar to “mood prone to changes”. Also, in item 10 of the questionnaire, the word “uncoordinated” was replaced with “I am less able to control my movements” in order to clarify the meaning of this statement. Similar adaptations were introduced throughout the document so that the items can be understood by persons of all backgrounds speaking Serbian and avoid misunderstandings. For further information, please contact the MAPI Research Trust (Link: https://eprovide.mapi-trust.org/instruments/fatigue-impact-scale, accessed on 16 August 2024).

The study was conducted at the Neurology Department of the General Hospital Subotica, Serbia, between June and December 2013, as part of the IPA EU MULTSCLER 2013 project between the General Hospital Subotica, Serbia, and the Neurology Clinic of the University of Szeged, Hungary. The group of MS patients included patients aged 18 or older with a confirmed diagnosis of relapsing remitting MS (RRMS) according to the 2010 McDonald criteria [21] and stable disease during the past six months. There was no cut off value for age or duration of disease but patients with significant disability with EDSS exceeding 5.5 were excluded. Further, we excluded patients who had had a relapse or infection, or an episode of elevated body temperature during the previous month, or those who used fatigue-inducing drugs such as corticosteroids, benzodiazepines, fampridine and other agents affecting central pathways. Also, we excluded patients who may have displayed fatigue secondary to any other systemic condition. 

The control group consisted of healthy volunteers who were matched according to age, gender and educational level. All participants of both groups were asked to sign a written informed consent. The study was approved by the Institutional Review Board of the General Hospital Subotica, Serbia (01-648/1/13).

The participants were asked to complete the FIS on two separate occasions three months apart. It was confirmed that the above-described inclusion and exclusion criteria still applied in the retest session, especially the absence of relapse, as well as progressive disease and use of the aforementioned drugs. There are certain factors that have been associated with increased risk of developing fatigue or increase severity of preexisting fatigue. In order to minimize the possible effect of interfering factors such as depression and progressive disease course with advanced disability, and in an attempt to isolate fatigue, all patients completed the Beck’s Depression Inventory (BDI) [22]. Furthermore, all participants were examined and physical disability was objectively quantified from 0 (no disability) to 10 (death from MS) using the Expanded Disability Status Scale (EDSS) [23]. 

### Statistical Analysis

Statistical analysis included the use of the *t*-test and the χ^2^-test depending on the type of data distribution. At first, an independent samples test (*t*-test for equality of means) was used, while an adjusted model was applied as Levene’s test for equality of variances showed heteroscedasticity, *p* < 0.01 for all of the comparisons. As the SD values were relatively high when compared to mean values, the non-parametric Mann–Whitney U test was also applied. The difference between MS patients and HC in total FIS scores and the subscale scores after eliminating depression indicated by BDI scores was investigated using covariance analysis (ANCOVA). The reliability of scores was assessed using intraclass correlation coefficients (ICCs) based on average measures, and the internal consistency was measured using Cronbach’s alpha and item-to-total correlations. Spearman correlation was used to determine the relationship between EDSS and FIS scores. The data were analyzed using the SPSS 20.0 software package.

## 3. Results

A total of 121 MS patients and 122 HC participated in this study. All participants completed both questionnaires fully on both occasions. The demographic and clinical data of MS patients and the HC group are presented in Table 1. They were matched with respect to age, gender and educational level, i.e., no statistically significant differences were found in mean age (t = 0.013, *p* = 0.990), gender (χ^2^ = 0.017, *p* = 0.896), educational level (χ^2^ = 5.462, *p* = 0.141) or marital status (χ^2^ = 0.391, *p* = 0.942) between participants of the two groups.

Basic data on results of the first (test) and second (retest) sessions, including total and subscale scores of FIS before and after eliminating depression, are shown in Table 2. Significantly higher scores were recorded in all three subscales (cognitive subscale *p*_1_(t) < 0.01, physical subscale *p*_1_(t) < 0.01, social subscale *p*_1_(t) < 0.01) and in total in both test and retest sessions (FIS_1_: *p*_1_(t) < 0.01 and FIS_2_: *p*_2_(t) < 0.01) for MS patients compared to HC.

Mean BDI scores were statistically higher for MS patients compared to HC subjects in both the first (*p*_1_(t) < 0.01) and the second (*p*_2_(t) < 0.01) session.

The non-parametric Mann–Whitney U test showed that the resulting significance levels also revealed higher values for subscale score mean values and total fatigue score values among the MS patients compared to the HC subjects in both the first (every *p*_1_(U) < 0.01), and second session (every *p*_2_(U) < 0.01). The same was found for BDI_1_ and BDI_2_.

After the elimination of depression, the differences were statistically significant in the first as well as in the second session for the cognitive subscale (*p*_1_(c) = 0.028 and *p*_2_(c) = 0.027), for the physical subscale (*p*_1_(c) < 0.01 and (*p*_2_(c) < 0.01) and for the social subscale (*p*_1_(c) < 0.01 and *p*_2_(c) < 0.01).

The reliability of scores was assessed using ICCs based on average measures. The internal consistency was measured using Cronbach’s alpha and item-to-total correlations. Results were obtained for all subjects of both MS and HC groups combined, for the subscales and the total FIS scale. Excellent reliability and mutual consistency of elements were proven for all three subscales and total scores, as can be seen in Table 3.

The significance of statistical differences between the MS patients and HC subjects was compared separately for each of the 40 items of the FIS. It was shown that for every single item, the average FIS score was statistically higher for the MS patients compared to the HC subjects in the first session (every single *p*_1_(t) < 0.01) and in the second session (every single *p*_2_(t) < 0.01). Almost all items of BDI have statistically significantly higher scores for MS patients in the first (*p*_1_(t) < 0.05, some *p*_1_(t) < 0.01) and in the second session (*p*_2_(t) < 0.05, some *p*_2_(t) < 0.01). Non-significant differences of BDI scores for MS patients were only found for two items in the first session, i.e., item B8 (*p*_1_(t) = 0.178) and item B12 (*p*_1_(t) = 0.088). In the second session, the only difference that was not significant was for item B9 (*p*_2_(t) = 0.109). 

Large item-to-total correlations were shown with most of the correlations being ≥0.8, indicating that the questionnaire is internally consistent, as can be taken from Table 4.

Methods of paired-samples statistics revealed significant correlations between scores of the first session (test) and the second session (retest). This applies to every single item (level of significance of correlation coefficients for every item is <0.01). Scores of cognitive subscale, physical subscale and social subscale of the first session are highly correlated with the respective scores of the second session (for every subscale *p* < 0.01). When observing total FIS score, the results of the first session and the second session are mutually highly correlated (*p* < 0.01). Scores for BDI_1_ and BDI_2_ are also highly correlated (*p* < 0.01).

The comparisons of mean scores of FIS_1_ and FIS_2_ for MS patients show insignificant differences for most of the items (*p* > 0.05), but there are significant differences between the first and the second session for seven items. Five of these items belong to the physical subscale: item 10 (translation of “I am more clumsy and uncoordinated”), (*p* = 0.002); item 14, (translation of “I am less motivated to do anything that requires physical effort”) (*p* < 0.01); item 31 (translation of “I am less able to complete tasks that require physical effort”), (*p* = 0.041), item 37 (translation of “I have to limit my physical activities.”), (*p* = 0.030); and item 38 (translation of “I require more frequent or longer periods of rest”), (*p* = 0.006). Item 18 (translation of “I find it difficult to make decisions”) (*p* = 0.044) belongs to cognitive subscale and, item 29 (translation of “I engage in less sexual activity”) (*p* = 0.048) belongs to social subscale. There is a significant difference between mean physical subscale scores for MS patients in the first and the second session (*p* = 0.024); the mean score was significantly higher in the first session compared to the second session.

The disability scores of MS patients determined by EDSS displayed statistically significant associations with the total FIS score, the cognitive, physical and social subscales and with BDI at both test and retest sessions. The corresponding Pearson correlation coefficients are presented in Table 5.

## 4. Discussion

Fatigue is one of the most frequent complaints of persons with MS. This primary fatigue occurs outside of relapse activity or muscle weakness and is thought to be initiated and sustained by a number of different factors. Fatigue significantly reduces quality of life in these patients [6,16]. A number of different scales and tests aim to adequately describe different aspects of fatigue [7,24]. 

The FIS in particular was developed to quantify aspects in three dimensions that are thought to underlie fatigue in 40 items. They are split to explore the effect of fatigue on various elements from different areas. Ten questions examine the physical domain, i.e., feelings of weakness, pacing and ability to perform physical activities as well as the need for periods of rest. The 10 questions that pertain to cognitive functioning inquire about motivation, concentration and thinking amongst others, while 20 items ask about social functioning. These include the effect of fatigue on isolation, workload, planning of and/or engaging in various activities, and functioning within a society or a community, e.g., family. The principal aim of the FIS is to improve our understanding of the way this complex symptom affects the patients’ health related quality of life and to provide insights into their functional status by asking the patients to subjectively grade different functional impairments in the past month [7,14,19]. 

The FIS has been previously validated for the use in MS patients mainly, but not exclusively, in several countries including Canada, the United States, France, Sweden, Hungary, Turkey and Russia [25,26,27,28,29,30]. In the past, this scale has also been validated for use in patients with other systemic conditions, e.g., chronic liver diseases [31,32].

Fatigue in MS is considered to be a multidimensional symptom that significantly alters an individual’s day-to-day functioning. In research settings, as well as in clinical routine, it is beneficial to understand the individual domains affected in each case. As for the plausibility of the scale, it was agreed upon that the FIS covers all important areas that need to be considered in the adaptation phase. 

During the translation process, several items had to be reviewed in the Serbian version. We suggest that the most notable and essential adaptation was in regards to the word “fatigue”. In some languages, such as German, the use of the English word “fatigue” has become partially accepted in certain settings [31]. On the other hand, in Serbian, there are two similar words, of which one was thought to be better fitting and was, therefore, chosen for all items. Similar smaller adaptations were introduced throughout the document so that it can be understood by Serbian-speaking persons of all backgrounds. 

Besides language, it is important to consider cultural factors. Differences in FIS subscores and total scores of patients in different countries may be attributed to vastly different cultural backgrounds, as seen in some studies [14,27,30]. In our cohort, all domains differed significantly between the MS and HC groups at the test and retest sessions, and such cultural differences were not detected in this study. 

Some studies found a positive correlation between FIS scores and depression [4,33,34,35]. Also, it has been shown that different scales show different degrees of association with scores of other health determinants, e.g., depression [36]. Still, after the elimination of depression, the FIS scores and subscores were significantly higher in the MS patients compared to HC subjects. 

When comparing total and subscale scores, they were found to be higher in the MS group compared to the healthy controls at both the test and retest sessions. Higher mean values on both total and subscale scores were observed in patients with MS compared to participants of the HC group after applying the non-parametric Mann–Whitney U test. The same was true for the BDI scores. Further, in this study we found a significant positive correlation between EDSS and total FIS, as well as between EDSS and the subscale scores. Similar findings have been reported in some other studies [13,35,37], although this was not demonstrated in all reports [26,33,38].

The reliability of scores was assessed using ICCs based on average measures, and the internal consistency was measured using Cronbach’s alpha and item-to-total correlations. Similar to studies conducted in other countries [28,30], excellent reliability and mutual consistency of elements were proven for all three subscales and the total scores. The total and subscale scores were also shown to be significantly correlated in both the test and retest sessions, indicating good test–retest reliability. 

This study included a representative sample of patients with RRMS; hence, it validates the use of the Serbian version of the FIS in this population. Patients with clinically active disease and non-ambulatory patients were excluded in order to minimize the effect of disease activity and progression as well as physical disability on the results. Using an appropriate tool to adequately describe fatigue and its effect on functionality is of key importance. This is especially true when clinicians depend on subjective reports. Using independent translation and back-translation, as well as taking into account suggestions from participants, ensures that the scale is well understood. It has also been described previously that these problems may be difficult to grasp and to describe due to the fact that they do not appear with certain characteristics that would be typical of MS [7].

A possible disadvantage of this study is the rather short time interval of three months between test and retest sessions. While fatigue is a chronic complaint that is not expected to change significantly during a period of three months, the interval was kept rather short in an attempt to minimize the potential risk of significant changes in the participants’ status. These changes could include a relapse during the said interval or the introduction of new medications, including corticosteroids, sedatives, amphetamines or fampridine. Finally, a change in the participants’ intent to take part in the project needs to be considered during that period.

## 5. Conclusions

In this study, we translated the FIS and investigated its psychometric properties in a selected group of patients with RRMS and matched healthy controls. The translated and adapted version of the FIS in Serbian language was shown to have good validity and reliability. Therefore, it was proven that the Serbian FIS can be reliably used as the clinical instrument of choice for the assessment and measurement of all three dimensions of fatigue and its impact on functionality in the target population of Serbian speaking patients with RRMS, regardless of their background.

## Figures and Tables

**Table 1:** Demographic and clinical data of MS patients and HC subjects.

	MS Patients	HC Subjects
Frequency (N)	121	122
Age (mean ± SD), years	45.26 ± 11.22	45.24 ± 11.27
Gender		
Female	98 (81%)	98 (80%)
Male	23 (19%)	24 (20%)
Educational status		
Elementary school	18 (15%)	18 (15%)
Middle school	68 (56%)	73 (60%)
High school	11 (9%)	3 (2%)
University	21 (18%)	27 (22%)
Not indicated	3 (2%)	1 (1%)
Marital status		
Single	21 (17%)	20 (16%)
Married	75 (62%)	80 (66%)
Divorced	14 (12%)	14 (11%)
Widowed	10 (8%)	8 (7%)
Not indicated	1 (1%)	0 (0%)
Disease duration (years)	11.80 ± 9.45	
EDSS	3.00 ± 2.08	

MS patients—multiple sclerosis patients, HC subjects—healthy control subjects.

**Table 2:** Validity of the FIS before and after the elimination of the effect of depression using parametric and non-parametric statistical methods.

	MS Patients Mean ± S.D.	HC Subjects Mean ± S.D.	N (MS/HC)	*t*-Test (With Depression)	Mann–Whitney U test (with Depression)	Covariance Analysis (After Eliminating Depression)
Test				*p*_1_(t)	*p*_1_(U)	*p*_1_(c)
Cognitive subscale_1_	10.9 ± 10.7	4.1 ± 5.8	116/117	<0.01	<0.01	0.028
Physical subscale_1_	15.9 ± 11.5	4.1 ± 5.8	113/117	<0.01	<0.01	<0.01
Social subscale_1_	23.4 ± 20.6	6.5 ± 9.8	101/109	<0.01	<0.01	<0.01
Total (FIS_1_)	48.4 ± 41.1	14.0 ± 19.6	96/105	<0.01	<0.01	<0.01
BDI_1_	11.3 ± 11.2	4.2 ± 4.8	107/117	<0.01	<0.01	-
Retest				*p*_2_(t)	*p*_2_(U)	*p*_2_(c)
Cognitive subscale_2_	9.7 ± 9.8	2.8 ± 4.3	117/118	<0.01	<0.01	0.027
Physical subscale_2_	14.6 ± 11.1	3.0 ± 4.5	121/122	<0.01	<0.01	<0.01
Social subscale_2_	22.5 ± 20.7	5.7 ± 8.4	102/116	<0.01	<0.01	<0.01
Total (FIS_2_)	45.3 ± 40.3	11.3 ± 16.7	100/113	<0.01	<0.01	<0.01
BDI_2_	10.6 ± 10.2	3.1 ± 4.0	108/116	<0.01	<0.01	-

MS patients—multiple sclerosis patients, HC subjects—healthy control subjects; Index 1: results for the first session (test); index 2: results for the second session (retest); N (MS/HC): Number of multiple sclerosis patients/number of healthy control subjects; *p*(t): level of significance for *t*-test, without eliminating depression; *p*(U): level of significance for Mann–Whitney U test, without eliminating depression; *p*(c): level of significance for covariance analysis after eliminating depression.

**Table 3:** Reliability of the Fatigue Impact Scale.

Variable	α	ICC
Cognitive subscale	α = 0.974	0.966
Physical subscale	α = 0.980	0.974
Social subscale	α = 0.990	0.980
Total scores	α = 0.990	0.990

α—Cronbach’s alpha; ICC—intraclass correlation coefficient.

**Table 4:** Fatigue Impact Scale item-specific statistics.

Item Number	Mean Score	Corrected Item-Total Correlation	Item Number	Mean Score	Corrected Item-Total Correlation
**Cognitive subscale**			**Social subscale**		
1	29.67	0.704	2	29.86	0.772
5	29.68	0.805	3	29.53	0.829
6	29.77	0.825	4	29.75	0.751
11	29.70	0.726	7	29.44	0.884
18	29.88	0.868	8	29.70	0.881
21	29.84	0.831	9	29.68	0.835
26	29.75	0.844	12	29.52	0.787
30	29.79	0.849	15	29.56	0.859
34	29.73	0.819	16	29.77	0.858
35	29.66	0.819	19	29.84	0.846
**Physical subscale**			20	29.76	0.895
10	29.53	0.907	22	29.62	0.852
13	29.46	0.859	25	29.56	0.879
14	29.42	0.866	27	29.63	0.904
17	29.46	0.882	28	29.70	0.827
23	29.39	0.886	29	29.64	0.784
24	29.55	0.892	33	29.87	0.820
31	29.37	0.888	36	29.72	0.877
32	29.99	0.678	39	29.81	0.828
37	29.42	0.890	40	29.68	0.892
38	29.41	0.888			

**Table 5:** Pearson correlation coefficients between the EDSS scores of MS patients and related variables.

Variable	First Session	Second Session
Cognitive subscale	0.440	0.360
Physical subscale	0.625	0.571
Social subscale	0.555	0.541
FIS	0.562	0.523
BDI	0.491	0.492

Significance for every item *p* < 0.01.

## Data Availability

The data from this study are available on request from the corresponding author. The data are not publicly available due to privacy and ethical restrictions.
